# Growth, health aspects and histopathology of brown bullhead (*Ameiurus nebulosus* L.): replacing fishmeal with soybean meal and brewer’s yeast

**DOI:** 10.1038/s41598-020-57722-3

**Published:** 2020-01-24

**Authors:** Daniel Matulić, Josip Barišić, Ivica Aničić, Tea Tomljanović, Roman Safner, Tomislav Treer, Jian Gao, Ines Glojnarić, Rozelindra Čož-Rakovac

**Affiliations:** 1 Department of Fisheries, Apiculture, Wildlife management and special Zoology, Faculty of Agriculture, University of Zagreb, Zagreb, Croatia; 2 Laboratory for biotechnology in aquaculture, Ruđer Bošković Institute, Zagreb, Croatia; 3 College of Fisheries, Huazhong Agricultural University, Wuhan, China; 4 Fidelta Ltd., Zagreb, Croatia

**Keywords:** Physiology, Ecology

## Abstract

A ten-week feeding trial was carried out to investigate the effects of replacing fishmeal (FM) with soybean meal (SBM) and brewer’s yeast (BY) on growth performance, blood parameters, oxidative stress and micromorphology of liver and intestines in brown bullhead (*Ameiurus nebulosus* L.). Fish were fed nine feeds in which FM was replaced with 25%, 50%, 75% and 100% SBM (K1, K2, K3 and K4) and 17% + 8%, 42% + 8%, 67% + 8% and 92% + 8% of SBM/BY combination (K5, K6, K7, K8). Growth indices showed greater outcomes for the K2 group in comparison to all other groups. A decrease in plasma cholesterol and triglycerides concentrations was found after FM replacement. Activity of SOD was higher in groups K4, K7 and K8. The early inflammatory indications with abnormal vacuolization of *lamina propria* and basal epithelium were present in diets K4 and K8. Hepatocytes were irregular in shape with signs of inflammatory reaction in diet K8. A decreased perimeter of hepatocyte nuclei was detected in all experimental diets when compared with the control. This study demonstrates that the optimal replacement of FM with SBM/BY in brown bullhead diets contains up to 50% of FM replaced with SBM in order to obtain advantageous growth performance and adequate health condition.

## Introduction

Aquaculture is one of the fastest growing industries in the food sector. The global supply of fish for human consumption has outpaced population growth in the past five decades - preliminary estimates suggest per capita intakes higher than 20 kg (20.2)^1^, double the level of the 1960s - that is mainly due to aquaculture growth, as the capture fisheries have stagnated in the last few decades. However, in 2010 aquaculture used 73% of global fishmeal production^2^. Therefore, Hardy^3^ argued that the industry would soon run out of sufficient quantities of fish oil and fishmeal (FM). As a result of increasing demand, limited supply and a dramatic increase in FM price, suitable alternative protein sources for fish feed have recently been intensively studied^4^. In modern intensive aquaculture, feed formulations may account for more than 50% of the total production costs. Any reduction in feed costs with preserved health status of fish is bound to have a direct positive effect on the profitability of aquaculture production^5–7^. Increasing feed efficiency, especially by improving the metabolic assimilation of dietary nutrients, is of the highest priority in contemporary animal production. The use of protein sources of plant origin as FM replacement in fish feed is a growing trend in the aquaculture industry. In this context, among commercially available plant protein alternatives, de-hulled and extracted SBM has one of the best amino acid balances^8^, except for the low level of methionine^9^. It is widely available at competitive prices^3^, and is therefore one of the main dietary protein alternatives to FM in the feed of aquacultured species^10–13^. Potential problems in soybean feeding exist due to the existence of antinutritional factors, which can cause malnutrition and lower palatability^14–16^. Several studies have demonstrated that the use of soy products in fish diets could lead to an inflammatory state of intestinal mucosa^17–21^. Morphological changes depend on the tolerance of the species, levels of inclusion of soy^22^ and different technological processes involved^23^. Thus, it is of critical importance to estimate the largest share of soy bean protein in fish feed for different fish species in aquaculture in order to avoid potential health issues, and at the same time to secure optimal growth performance. External factor, such as quality of diet, would affect the health condition of fish^24^. Oxidative stress markers and the levels of different constituents in plasma have been used as indices for evaluating the physiological and health condition of fish^25–27^. Brewer’s yeast (BY) represents a single-cell protein source (SCP) and can easily be cultured and obtained in a pure form to be used in aquaculture as a feed ingredient. These non-conventional alternative protein sources are frequently used as feed ingredients for fish because of their extremely rich and high quality vitamin and mineral complex (especially vitamin B complex) and amino acid composition. BY is considered a quality immunostimulant^28^, as well as FM protein alternative in fish feeds^29–33^. Histopathological analysis of fish tissues has been used as a biomarker in assessment of internal effect (nutrition) and external (aqueous environment) conditions^34–36^. Histological studies of the digestive system are considered to be a good indicator of metabolic, health and nutritional status of fish^21,37,38^. The liver function includes metabolism of proteins, lipids and carbohydrates, and is an important organ for the storage and distribution of compound reserves. On the other hand, the main function of intestines is related to the absorption of nutrients and water. Monitoring of the histological structure of fish liver and intestines is already recognised as a method of choice for assessing the effects of nutrient mixtures which use raw materials of plant origin^39^. Catfish (*Ictalurus spp*.) is the dominant aquaculture species in the United States, far exceeding farm-raised trout, salmon, tilapia, crayfish and shrimp in both volume and value^40–42^. Brown bullhead is a member of the family Ictaluridae, an omnivorous and benthic fish species which have been utilized within their native and introduced range primarily for sport and aquaculture, although the species may also be found inadvertently within the aquarium trade. Economic benefits from aquaculture occur primarily in Chile, China, Bulgaria and Belarus^43–46^ although the magnitude of these benefits remains uncertain. SBM and BY have been widely used in livestock nutrition and several studies have assigned them as a protein source in fish feed^30,31,47,48^. To our knowledge, this is the first study on FM replacement with implications on growth and digestive system morphology in brown bullhead. Thus, the purpose of this study was to evaluate the effects of FM replaced with SBM and a combination of SBM/BY in the diet of brown bullhead by means of assessing the basic growth and health parameters, micromorphology and qualitative histopathology of liver and intestinal tissue, with the aim of highlighting the optimal SBM and BY share in fish feed for the cultured brown bullhead.

## Material and Methods

### Fish and feeding trial

Brown bullheads were obtained from Pakračka Poljana, a commercial fish farm (N45°30′32.79″, E16°56′07.09″). Prior to the experiment, the fish were acclimated to the experimental conditions for 8 weeks during which they were fed the control diet (K0). Two hundred and seventy brown bullheads, with an initial average weight of 120 g, were then randomly distributed in twenty-seven 115 L rectangular tanks (10 fish per tank) and inserted into a partially recirculating system (RAS) (10% of daily water renewal) for 10 weeks. Nine experimental diets in three groups were randomly assigned within the 27-tank system. Low-pressure electrical blowers provided aeration via air stones while dissolved oxygen (DO) levels were maintained at the average of 4.83 mg L^−1^. Daily amounts of dissolved oxygen and pH value (7.41–7.80) were measured by HI 2020-01 edge^®^ probe. Total ammonia nitrogen NH_3_-N (0.09 mgL^−1^ – 0.69 mgL^−1^), nitrite NO_2_^−^ (0.04 mgL^−1^ – 0.22 mgL^−1^) and nitrate NO_3_^−^(0.00–12.70 mgL^−1^) were measured using HI 83200 spectrophotometer. Tap water was filtered out with a filter system (size: h = 1000 mm, ø = 300 mm), consisting of magnetic pipes, aquarium sponge, ceramic cylinders, synthetic fibres and granular active carbon. The whole system shared the same water at a flow rate of 1.5 Lmin^−1^ (exchange rate of 10% of the system volume per day) and each tank was supplied with primary biological filter. Water temperature was measured daily and maintained at 20.0 ± 0.43 °C. During the experimental period, the fish were subjected to a 11:13 h (light:dark) photoperiod under fluorescent lightning. The guidelines laid down in Directive 2010/63/EU on the protection of animals used for scientific purposes were followed during the research. A special care was taken regarding part 11 (Fish) of the Directive (water supply and quality, stocking density and environmental complexity, feeding and handling and killing the fish). The research was carried out at Department of Fisheries Lab and the experimental protocol was approved by Ethical committee of University of Zagreb Faculty of Agriculture (Class: 114–04/19-03/08; Reg. Nr. 251-71-29-02/11-19-2).

### Experimental diets

The basic mixture for the production of concentrated complete feed was based on the nutritional needs for Ictaluridae^7,49,50^. Nine isonitrogenous (28.7% crude protein) and isoenergetic (17.3 MJ kg^−1^ GE) feeds were containing 0 (K), 25% (K1), 50% (K2), 75% (K3) or 100% (K4) of FM replaced with SBM and 17% + 8% (K5), 42% + 8% (K6), 67% + 8% (K7) and 92% + 8% (K8) of a combination of SBM and BY. The control feed (K) contained 30% of FM. (Table 1). Feeding rate and the protein percentage utilized in the diet were optimum for the growth of *Ictalurus punctatus* adults, considering water temperature and fish age in the study^49^. The diets were processed by blending the dry ingredients into a homogenous mixture and the mixture was passed through a laboratory pellet mill at 2.5 mm diameter at Nutrient d.o.o. in Zagreb, Croatia. Triplicate groups of fish were hand-fed once a day (at 04:00 p.m.) in the total quantity of 1% of live body mass for 10 weeks.

**Table 1:** Ingredients and proximate composition of the experimental diets.

Diets	K0	K1	K2	K3	K4	K5	K6	K7	K8
***Ingredients (g kg***^**−*1***^ ***as feed basis)***									
Fish meal (600 g/kg protein)	300	225	150	75		225	150	75	
SBM (490 g/kg protein; defatted, toasted)		75	150	225	300	51	126	201	276
Inactive BY (400 g/kg protein)						24	24	24	24
Rapeseed meal	250	250	250	250	250	250	250	250	250
Corn meal	110	180	210	230	180	220	200	230	210
Wheat meal	290	220	190	170	220	180	200	170	190
Soybean oil	10	10	10	10	10	10	10	10	10
Vitamin-mineral mix ^¥^	20	20	20	20	20	20	20	20	20
Binder (Neubabonder 100)	20	20	20	20	20	20	20	20	20
***Proximate composition*** **(g kg**^**−1**^ **of diet)**									
Crude protein	285.4	280.0	282.5	297.2	278.6	298.2	287.8	290.5	284.9
Crude lipid	50	41	45	36	31	45	42	37	32
Crude fiber	34	38	36	42	41	39	37	41	37
Ash	92	70	77	56	40	75	66	56	41
Dry matter	867	852	843	873	805	868	845	842	823
NFE	405.6	423.0	402.5	441.8	414.4	410.8	412.2	417.5	428.1
GE (MJ kg^−1^)	17.45	17.34	17.29	17.25	17.41	17.35	17.36	17.3	17.31

K0, control diet; K1, 25% SBM; K2, 50% SBM; K3, 75% SBM; K4, 100% SBM; K5, 25% (17%SBM + 8%BY); K6, 50% (42%SBM + 8%BY); K7, 75% (67%SBM + 8%BY); K8 100% (92%SBM + 8%BY); NFE - nitrogen free extract (%NFE = %Dry matter-(% Protein + % Ash + % Lipid + % Fiber); GE – Gross Energy; ¥Vitamine – mineral mix provided the following: Vit.A, 2.000 000 IU; Vit.D3, 50 000 IU; Vit E (DL-α-Tocopherol), 5000 mg; Vit.K3 90 mg; Vit. B1, 100 mg; Vit.B2, 960 mg; Vit.B6, 1.000 mg; Vit. B12, 2 mg; Vit. B3, 1.400 mg; Pantothenic acid, 1.500 mg; Folic acid, 150 mg; Biotin, 50 mg; Vit. C, 5.000 mg; Inositol, 5.000 mg; Choline chloride, 40. 000 mg; Mn, 250 mg; Zn, 2.000 mg; Fe, 3.000 mg; Cu, 500 mg; I, 100 mg; Co, 10 mg; Se, 25 mg; Ca, 10 g; P, 3,5 g; Antioxidant BHA (E320) 10.000 mg; Carrier: Flower, up to 1.000 g; Origin of the feed ingredients: FM - Adria d.d., SBM – IREKS Aroma d.o.o., BY - Sladorana d.d., Rapeseed meal - Bio Uljarice d.o.o.

### Analytic methods and sampling

Proximate analyses of the diets were done by standard methods^51^. Crude protein (N x 6.25) was determined by the Kjeldahl method. Crude lipid was determined by the dichloroether extraction by Soxtec System HT. For dry matter analysis, feed was oven-dried for 24 h at 105 °C. For crude ash estimation, feed was incinerated at 550 °C in a muffle furnace for 24 h. At the end of the growth trial, 135 fish were euthanised (3 min in 100 mg L^−1^ MS-222) (Fluka, Sigma-Aldrich, Saint Louis, USA)^52^, weighed and dissected for hepatosomatic and viscerosomatic index calculation. For haematological and biochemical blood tests, 135 samples (15 from each feeding group) were taken, and for the analysis of oxidative stress 81 samples (9 samples from each feeding group) were taken. Hematological (hematocrit and hemoglobin) and biochemical (glucose, total protein, cholesterol, triglycerides, globulins AST, ALT and electrolytes [Na, Ca]) analyses were performed with an Olympus 400 AI analyzer. Glutathione reductase (GSH-Red), glutation peroxidase (GSH-Px), paraoxonase (PON) and superoxide dismutase (SOD) were determined in all blood samples (Randox, Ireland) on the SABA 18 biochemical analyzer (AMS, Italy). For microscopic studies, samples of liver and proximal intestine from 15 fish (5 fish per tank) from each dietary group were removed immediately after the fish had been euthanized and fixed in 10% neutral formalin. After fixation, portions of intestinal and liver segments were dehydrated in raising ethanol concentrations, cleared in xylene, embedded in paraffin blocks and sectioned to about 2–3 µm. The sections were then deparaffinised with xylene and rehydrated in decreasing concentrations of ethanol, stained with haematoxylin and eosin for histopathological examination (H&E), and with periodic-acid stain (PAS) for glycogen and goblet cell visualization. Slices were then washed in distilled water, dehydrated with increasing concentrations of ethanol, cleared in xylene and embedded in Canada balsam. Microphotographs of histological preparations were taken with a digital camera DP70 Olympus^®^ connected to an Olympus^®^ BX51 binocular microscope and analysed with Microsoft AnalySIS^®^ Soft Imaging System. Histological examination included major alterations from normal cell morphology. For intestinal micromorphological parameters, goblet cell diameters (µm) on 100 µm of intestine mucosa were measured by performing 50 measurements per animal (5000 µm fold length per animal). For morphometric analysis of liver condition, perimeters of hepatocyte nuclei (HN*p*, µm) in the middle of liver section (100 hepatocyte nuclei measurements per animal) were measured.

### Data collection – growth indices and morphological evaluation

Growth performance indices were calculated accordingly:

$${\rm{WG}}( \% )=\frac{{\rm{Final}}\,{\rm{weight}}\,-\,{\rm{Initial}}\,\text{weight}\,}{{\rm{Initial}}\,{\rm{weight}}}\times 100$$

$${\rm{FCR}}=\frac{{\rm{Dry}}\,{\rm{weight}}\,{\rm{of}}\,{\rm{feed}}\,{\rm{administered}}}{{\rm{Wet}}\,{\rm{weight}}\,{\rm{gained}}\,{\rm{by}}\,{\rm{the}}\,{\rm{fish}}}$$

$${\rm{SGRw}}( \% )=\frac{\mathrm{Ln}\,{\rm{Wt}}-\,\mathrm{Ln}\,\text{Wo}\,}{{\rm{days}}}\times 100$$

where LnWt = Napierian logarithm of total weight of fish at t days; Ln Wo = Napierian logarithm of initial total weight of fish.

At the end of the experiment, visceral condition was assessed by calculating the viscerosomatic index (VSI) as:

$${\rm{VSI}}=\frac{{{\rm{W}}}_{V}}{{{\rm{W}}}_{t}}\times 100,$$

where W_*V*_ is the weight of the fish’s viscera (g) and W_*t*_ is the total body weight (g).

Liver condition was calculated by the hepatosomatic index (HSI) as:

$${\rm{HSI}}=\frac{{W}_{L}}{{{\rm{W}}}_{t}}\times 100,$$

where W_*L*_ is the weight of the liver (g) and W_*t*_ is the total body weight (g).

Goblet cell density index (GCsi) as an indicator of intestinal tissue condition was calculated as:

$${\rm{GCsi}}=\frac{{\sum }^{}{{\rm{GC}}}_{{p}}}{100\,{\rm{\mu }}m\,IM}$$

where ∑GC_*p*_ is the sum of goblet cell perimeters and 100 µm *IM* presents 100 µm of intestinal mucosa.

### Statistical analyses

Treatments are assigned to experimental units completely at random. Data were analysed in a one-way analyses of variance ANOVA in order to determine the effect of treatments; Fisher’s LSD test followed in order to detect significant differences between the groups. The results were considered significant at *p* < 0.05. The software used was SPSS v.19.0^53^. Redundancy analysis (RDA) was used as a constrained linear ordination technique to correlate experimental diets as independent variables (dummy variables) with growth performance and health parameters, as dependent variables. The differences between variables were tested by Monte Carlo test with 499 permutations (*p* < 0.05)^54^. Canoco 4.5.5 for Windows was used for the analysis^55^ according to^54^.

## Results

### Growth performance

The initial TL (total length) was uniformed in all feeding groups and averaged 22.53 ± 1.06 cm. The final TL was significantly different between the groups (*p* < 0.05) with the highest values in the K5 feeding group (24.83 ± 1.11) (Table 2). The initial average individual weight of the fish was uniform and ranged from 125.94 ± 22.71 g (K2) to 132.7 ± 18.86 g (K7). A significant difference of final weight values was observed in K2 compared to the groups with 50 or higher percentage of FM replacement, regardless of the inclusion of BY. At the end of the trial, a significantly lower final weight was achieved by complete replacement of FM (SB + PK 100%) in the K8 group. The average values of weight gain (WG) at the end of the trial were in the range from the lowest (17.4 ± 2.90) in K8 up to the highest (33.24 ± 1.04) in K2. In comparison to other groups, significantly higher results of average WG values were indicated for the K2 group. Also, a significant difference was not observed between control (K0) and groups K1, K5 and K6. The average values of the feed conversion ratio (FCR) ranged from 1.49 ± 0.28 (K2) to 2.49 ± 0.4 for K8. The average specific growth rate SGRw values ranged from the highest (0.65 ± 0.12) in the K2 feeding group to the lowest (0.41 ± 0.07) in the K8 group. SGRw values indicate a significant seclusion of the K2 feeding group in comparison to other feeding groups. Along with K2, the groups K1, K5 and K6 did not significantly deviate from the mean values of the control feeding group K0. Significantly lower results were indicated by fish fed 75% of SBM (K3) and full substitution of FM with SBM and BY (K8).

**Table 2:** The average values of fish growth parameters during the feeding period (Mean ± SD).

Growth parameter	Experimental diets								
	K0	K1	K2	K3	K4	K5	K6	K7	K8
TL	24.47 ± 1.23^abcd^	24.66 ± 1.06^ab^	24.8 ± 1.33^ab^	24.29 ± 1.09^bcd^	24.18 ± 1.43 ^cd^	24.83 ± 1.11^a^	24.68 ± 1.42^ab^	24.54 ± 1.22^abc^	23.99 ± 1.32^d^
InW	127.91 ± 21.79	126.98 ± 17.78	125.94 ± 22.71	128.92 ± 19.22	129.45 ± 25.35	129.81 ± 23.71	130.95 ± 21.77	132.7 ± 18.86	129.86 ± 23.44
FW	191.57 ± 30.91^abc^	191.14 ± 27.57^abc^	198.1 ± 38.32^a^	174.61 ± 24.78^def^	180.77 ± 36.47^cdef^	194.78 ± 29.23^ab^	192.48 ± 30.64^abc^	184.13 ± 33.36^bcde^	171.68 ± 33.91 ^f^
WG	29.28 ± 0.98^bc^	28.01 ± 2.36^b^	33.24 ± 1.04^a^	19.31 ± 2.95^e^	22.02 ± 2.83^de^	28.85 ± 2.01^bc^	27.03 ± 1.58^cd^	20.74 ± 0.80^e^	17.4 ± 2.90^e^
FCR	1.67 ± 0.4^a^	1.65 ± 0.23^a^	1.49 ± 0.28^a^	2.28 ± 0.47^cd^	1.82 ± 0.37^ab^	1.67 ± 0.34^a^	1.76 ± 0.37^ab^	2.10 ± 0.38^bc^	2.49 ± 0.4^d^
SGRw	0.588 ± 0.08^ab^	0.59 ± 0.09^ab^	0.65 ± 0.12^a^	0.44 ± 0.1^d^	0.50 ± 0.11^c^	0.588 ± 0.11^ab^	0.558 ± 0.11^bc^	0.476 ± 0.08^cd^	0.406 ± 0.07^d^

TL – Total length; InW – Iinitial weight; FW – Final weight; WG – Weight gain; FCR – Feed conversion ratio; SGRw – Specific growth ratio (weight); The table shows values of mean ± SD of three experimental repetitions; Values within the same row with different superscript differ significantly (*p* < 0.05); Experimental diets are explained in the foregoing text.

### Health aspects

The results from hematological and biochemical analysis (mean ± SE) are summarised in Table 3 with different letters to indicate significant differences (*p* < 0.05) between the groups. In brief, plasma total protein and glucose levels did not differ among the groups but concentrations of cholesterol and triglyceride were significantly lower in all experimental groups with FM protein replacement when compared to control group. Activity of AST was highest in group K4. Concentration of sodium was increased in groups K3-K4 and K7-K8, while concentration of chloride was increased in all groups except K2. Concentration of globulin was decreased in groups K7 and K8. Activity of SOD was higher in groups K4, K7 and K8, while activity of GSH-red was lower in groups with 50% and higher replacement of FM. Other health markers were not statistically different between groups.

**Table 3:** The average values of hemoglobin, hematocrit, plasma biochemical parameters (n = 135) and oxidative stress markers (RANDOX) (n = 81).

Parameter	Experimental diets								
	K0	K1	K2	K3	K4	K5	K6	K7	K8
**HGL** (g l^−1^)	80 ± 15.44	66 ± 15.26	80 ± 10.67	69 ± 13.43	64 ± 18.54	60 ± 18.57	72 ± 17.60	79 ± 18.64	72 ± 23.48
**HTC** (g l^−1^)	35 ± 5.01	32 ± 6.52	35 ± 4.58	33 ± 7.65	34 ± 8.79	23 ± 11.92	29 ± 10.02	35 ± 5.09	33 ± 11.71
**T_Prot**. (g l^−1^)	41.8 ± 5.2	39.4 ± 4.5	39.5 ± 4.9	39.6 ± 4.3	41.0 ± 3.9	39.8 ± 5.3	38.9 ± 4.3	36.0 ± 4.4	37.3 ± 4.8
**Glob**. (g l^−1^)	30.6 ± 3.8^a^	28.5 ± 3.3^ab^	28.6 ± 3.6^ab^	28.5 ± 3.0^ab^	29.5 ± 2.6^a^	29.0 ± 3.9^ab^	28.4 ± 3.0^ab^	26.0 ± 2.9^b^	26.8 ± 3.6^b^
**Gluc**.(mmol l^−1^)	3.5 ± 1.0	3.7 ± 1.3	4.9 ± 1.5	3.9 ± 0.7	4.0 ± 1.1	4.0 ± 1.2	4.1 ± 1.0	4.0 ± 0.8	4.5 ± 1.2
**Cholest**. (mmol l^−1^)	5.4 ± 1.4^a^	3.6 ± 0.9^cd^	4.2 ± 0.9^bc^	3.9 ± 0.6^bcd^	3.8 ± 0.9^bcd^	4.5 ± 0.9^b^	3.8 ± 0.5^cd^	3.6 ± 0.9^cd^	3.5 ± 0.7^d^
**Tryglic**. (mmol l^−1^)	7.8 ± 2.2^a^	3.9 ± 1.2^de^	5.7 ± 1.6^b^	3.2 ± 1.0^de^	4.1 ± 1.3^cd^	5.6 ± 2.1^b^	4.1 ± 1.3^cd^	2.8 ± 1.0^e^	3.0 ± 0.9^de^
**AST** (U l^−1^)	211.1 ± 44.4^cd^	188.7 ± 49.4^d^	215.5 ± 45.7^cd^	278.6 ± 222.5^abc^	318.6 ± 149.9^a^	301.8 ± 107.7^ab^	233.4 ± 85.0^bcd^	273.9 ± 155.7^abc^	251.9 ± 114.4^abcd^
**ALT (**U l^−1^**)**	2.4 ± 1.1	1.8 ± 0.8	1.9 ± 0.8	2.1 ± 1.8	2.84 ± 1.2	2.80 ± 1.0	2.6 ± 1.3	2.4 ± 1.4	2.0 ± 1.1
**Na** (mmol l^−1^)	132.7 ± 1.5^e^	133.5 ± 2.3^cde^	132.7 ± 1.3^de^	135.0 ± 1.6^abcd^	136.9 ± 2.9^a^	134.6 ± 1.7^bcde^	134.6 ± 1.6^bcde^	135.9 ± 1.5^ab^	136.0 ± 2.3^ab^
**Cl** (mmol l^−1^)	106.9 ± 2.0^e^	110.6 ± 1.7^d^	108.3 ± 2.1^e^	111.8 ± 2.0^cd^	113.0 ± 2.0^bc^	111.6 ± 1.5^cd^	112.9 ± 1.3^bc^	113.6 ± 1.6^ab^	114.7 ± 1.8^a^
**GSH-Px**(U l ^−1^)	58715.6 ± 11841.4	54167.8 ± 17810.4	66476.7 ± 10254.1	41688.9 ± 15386.5	55751.1 ± 14037.2	47698.9 ± 15179.7	51714.4 ± 9933.2	48326.7 ± 13236.8	49783.3 ± 13982.5
**SOD** (U l ^−1^)	4061.3 ± 1960.6^bc^	4777.2 ± 2224.5^bc^	4582.8 ± 1080.7^bc^	3585.9 ± 1336.8^c^	6122.67 ± 3627.1^ab^	4813.8 ± 1771.1^bc^	4494.3 ± 1092.1^bc^	7382.1 ± 3370.8^a^	5703.8 ± 2460.12^ab^
**GSH-Red**(U l ^−1^)	290 ± 61.64^a^	260 ± 112.14^a^	156.67 ± 57.89^b^	156.67 ± 33.91^b^	158.89 ± 41.36^b^	268.89 ± 98.04^a^	162.22 ± 37.01^b^	186.67 ± 82.31^b^	184.44 ± 83.08^b^
**PON** (U l ^−1^)	1.16 ± 0.26	1.24 ± 0.27	1.04 ± 0.42	1.07 ± 0.38	1.48 ± 0.53	1.33 ± 0.60	1.56 ± 0.63	1.61 ± 0.48	1.67 ± 0.56

The table shows values of mean ± SD of three experimental repetitions; Values within the same row with different superscript differ significantly (*p* < 0.05); (K0-K8 – experimental diets; HGL – Hemoglobin; HTC – Hematocrit; T_Prot – Total proteins; Glob - Total globulins; Gluc – Glucose; Cholest – Cholesterol; Tryglic – Tryglicerides; AST - Aspartate transaminase; ALT – Alanine aminotransferase; Na – Sodium; Cl – Chloride; GSH-Px - Glutathione peroxidase; SOD - Superoxide dismutase; GSH-Red - Glutathione reductase; PON – Paraoxonase 1.

### Tissue morphology

**Figure 1:**
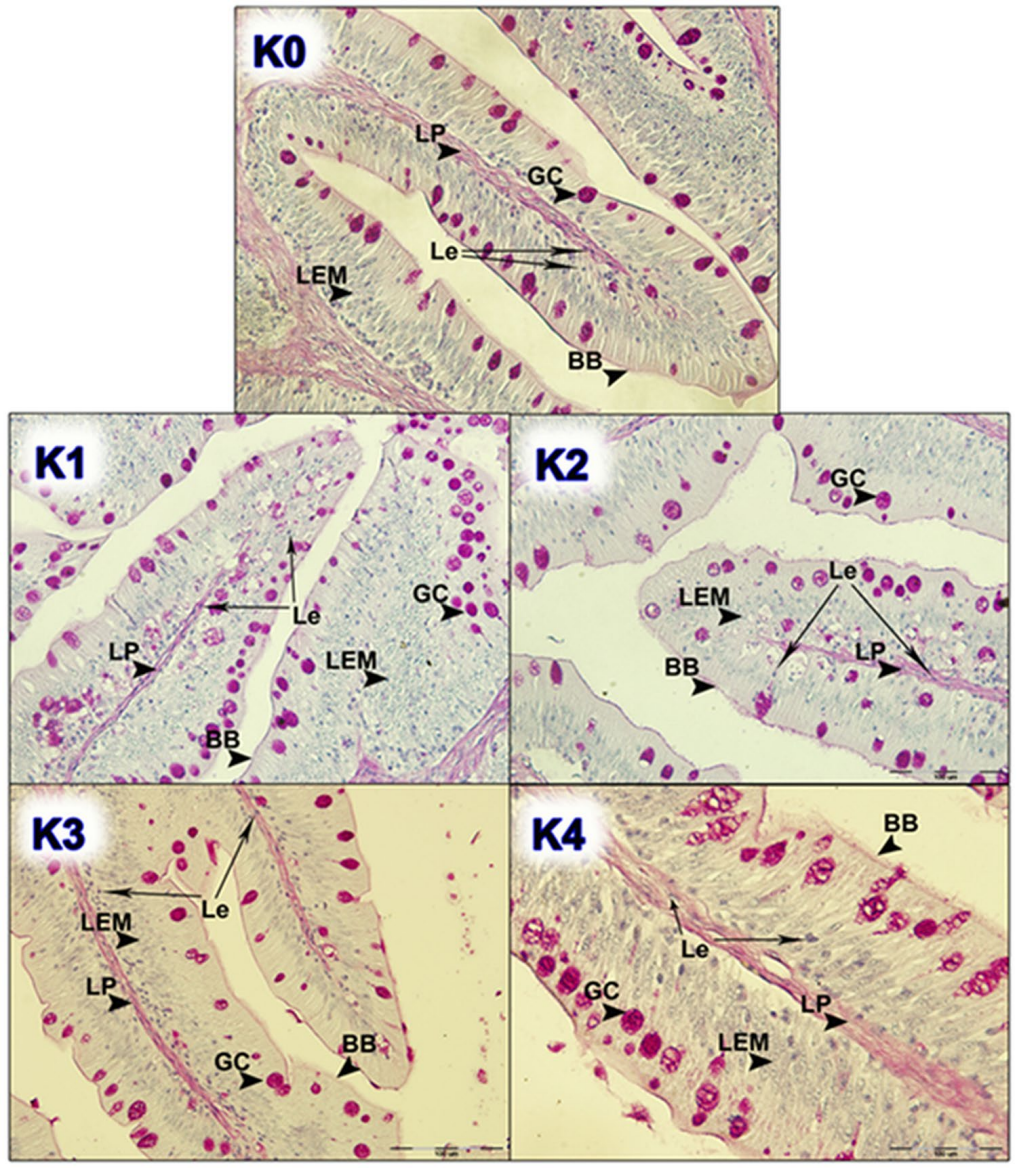
Histological examination of intestinal tissue of fish fed different dietary treatments (K1, K2, K3 and K4) in comparison with control group (K0); GC, goblet cells; LP, *lamina propria*; LEM, *lamina epithelialis mucosae*; Le, leukocytes; BB, Brush border cells. Scale bar: 100 µm.

**Figure 2:**
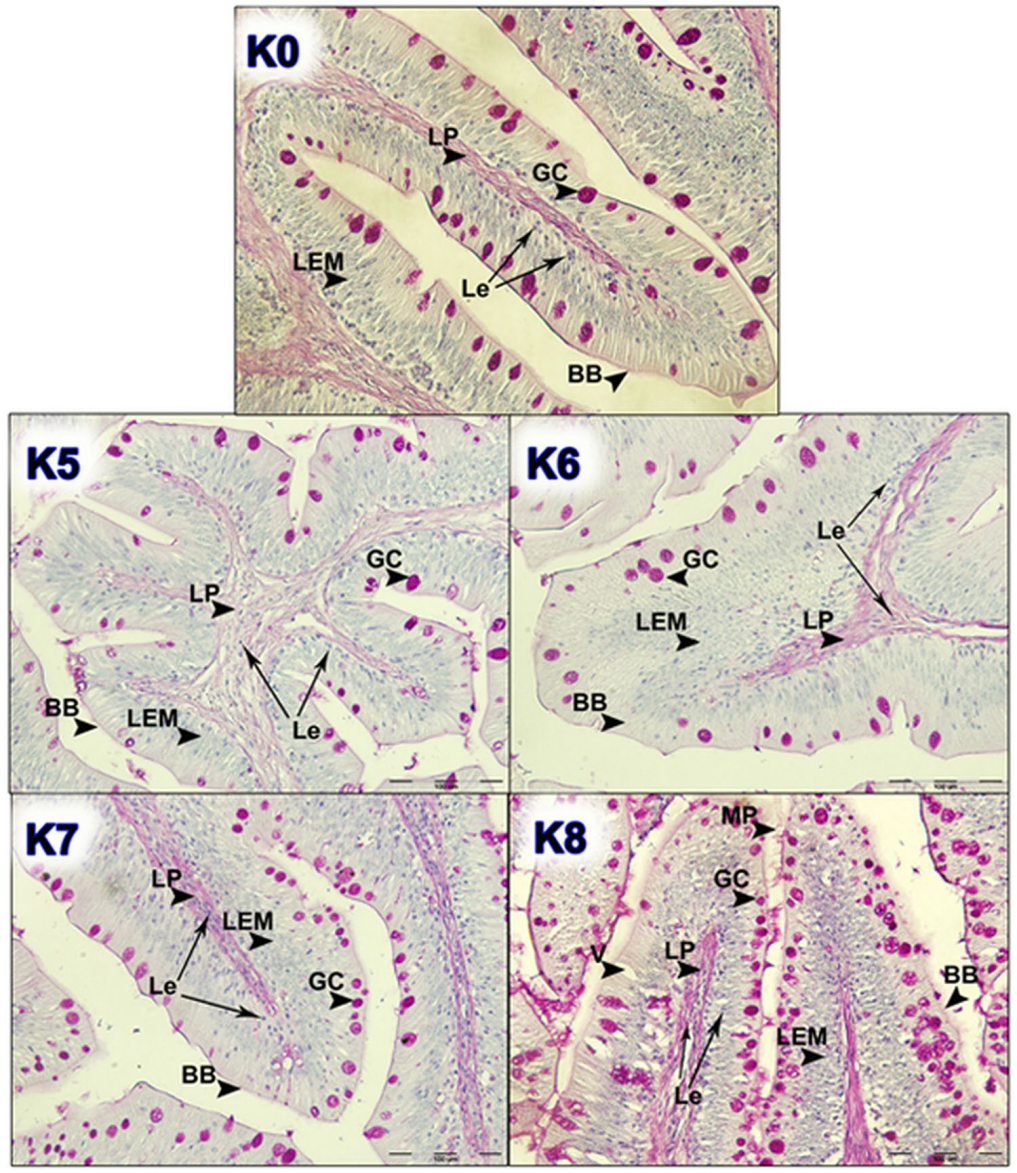
Histological examination of intestinal tissue of fish fed different dietary treatments (K5, K6, K7 and K8) in comparison with control group (K0); GC, goblet cells; LP, *lamina propria*; LEM, *lamina epithelialis mucosae*; Le, leukocytes; MP, accumulation of mucus; BB, Brush border cells. Scale bar: 100 µm.

**Figure 3:**
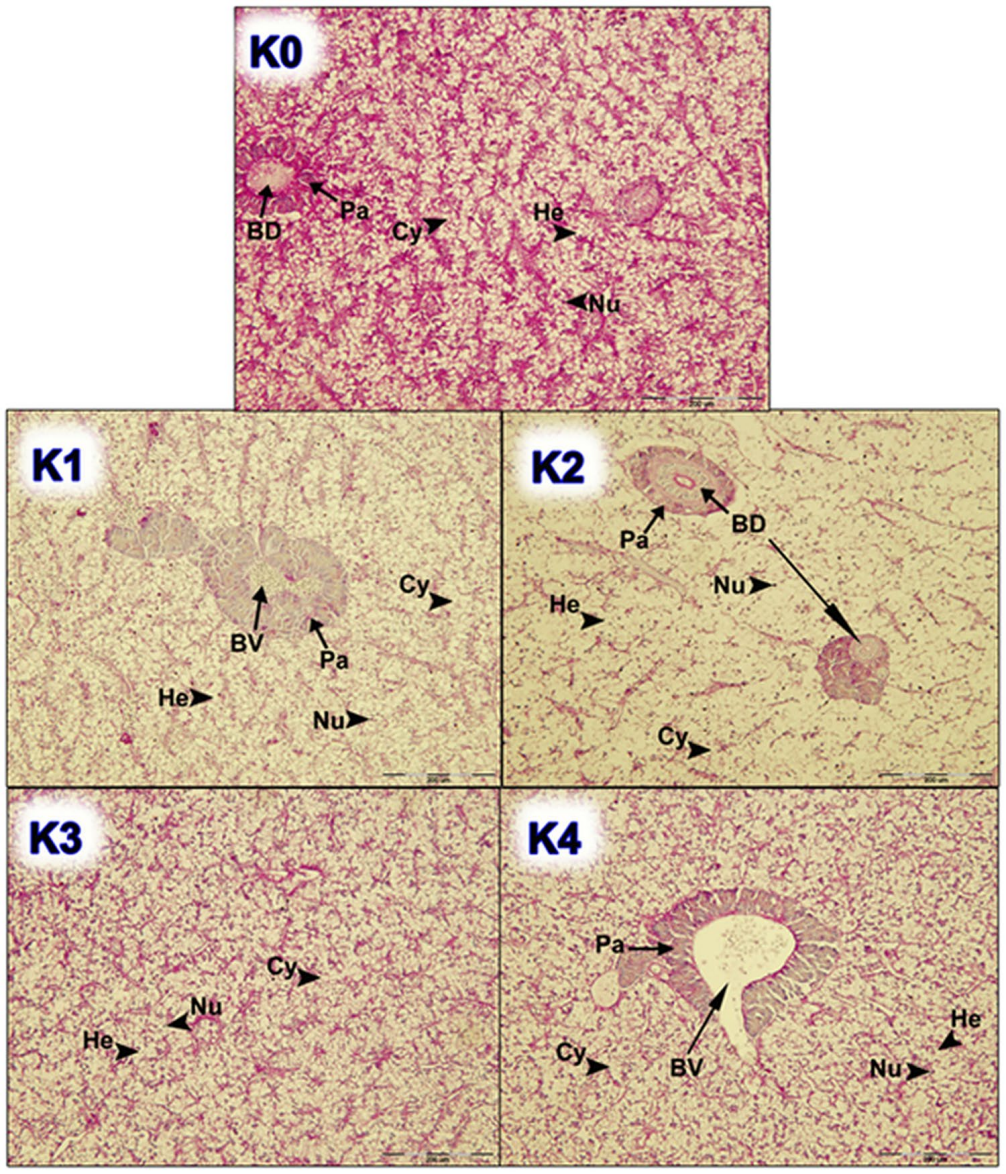
Histological examination of liver tissue of fish fed different dietary treatments (K1, K2, K3 and K4) in comparison with control group (K0); (He, hepatocyte; Nu, nucleus of hepatocytes; Pa, pancreas tissue; Cy, cytoplasm; BD, bile duct; BV, blood vessel). Scale bar: 200 µm.

**Figure 4:**
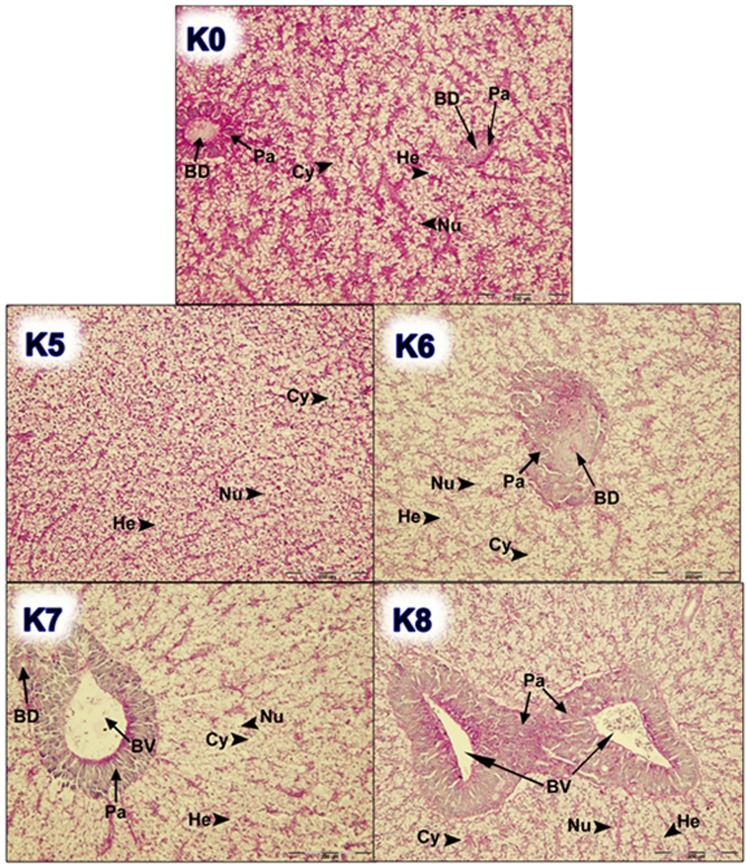
Histological examination of liver tissue of fish fed different dietary treatments (K5, K6, K7 and K8) in comparison with control group (K0); (He, hepatocyte; Nu, nucleus of hepatocytes; Pa, pancreas tissue; Cy, cytoplasm; BD, bile duct; BV, blood vessel). Scale bar: 200 µm.

In order to distinguish the effect between different dietary treatments of brown bullhead, histopathological analysis of intestinal and liver tissue from the fish fed SBM and SBM/BY as an alternative protein source was preformed and compared with the fish fed the control diet (Figs. 1, 2, 3 and 4).

**Figure 5:**
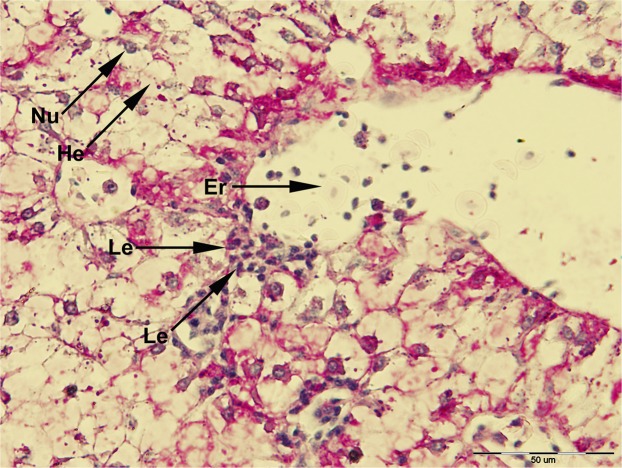
Histological examination of liver tissue of fish fed dietary treatment K7 (He, hepatocyte; Nu, nucleus of hepatocytes; Le, Leukocytes; Er, erythrocytes). Scale bar: 50 µm.

Appearance of brown bullhead intestinal folds showed high ramification and filled the entire intestinal lumen. Histopathological examination of the proximal intestine in fish originating from nutritional groups K0, K1, K2, K3 and K5 showed normal characteristics of *lamina epithelialis mucosae* and *lamina propria* (connective tissue), neatly defined by the ciliated epithelium and properly distributed goblet (PAS positive acidophilus coloured) cells. Histological examination of intestine micrographs from the feeding groups K4 and K8 indicated the initial inflammatory reactions, like hyperplasia of *lamina propria* and *lamina epithelialis*. For all diets, in larger or smaller proportions, cell infiltration with leukocytes in between the connective tissue and epithelium was observed. Leukocytes are visible as small egg-like cells with a dark-coloured core (Fig. 5).

**Figure 6:**
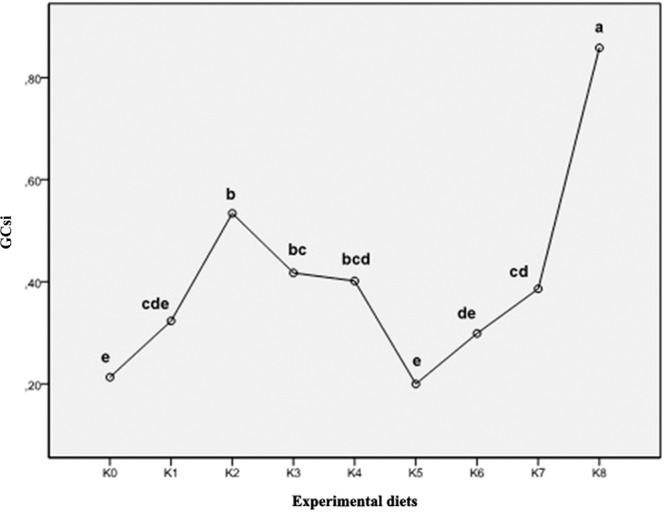
Index of goblet cell density of fish fed control and experimental diets (GCsi, Goblet cell density index).

In the feeding groups K6 and K7, an increase in the surface of *lamina epithelialis mucosae* was noted, while in the group K8 the presence of abnormal vacuolisation and increased number of irregularly distributed goblet cells is noted and confirmed by the analysis of the quantitative index of the surface of goblet cells (Fig. 6).

**Figure 7:**
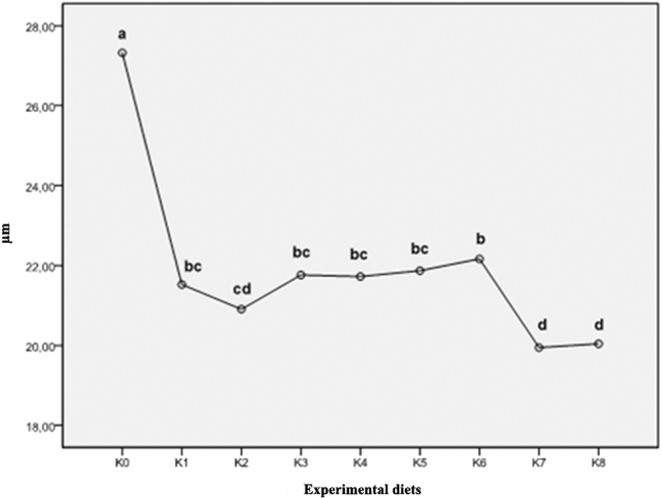
Hepatocytes nuclei perimeter size (µm) of fish fed control and experimental diets.

**Figure 8:**
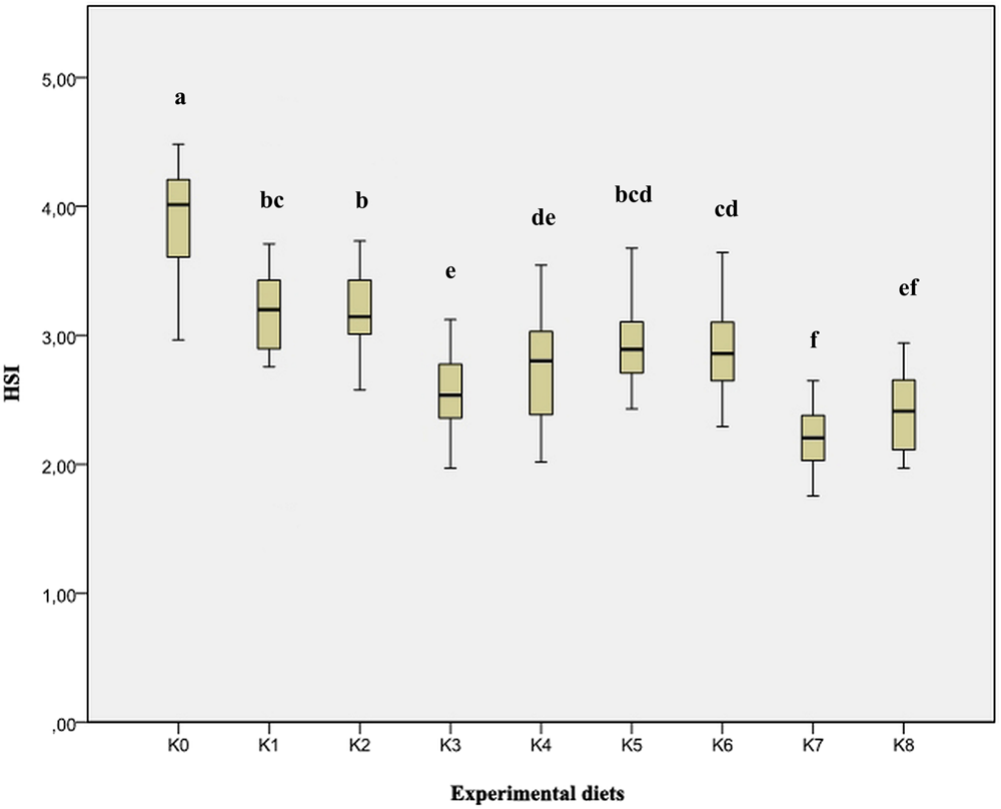
Hepatosomatic index (HSI) of fish fed control and experimental diets.

**Figure 9:**
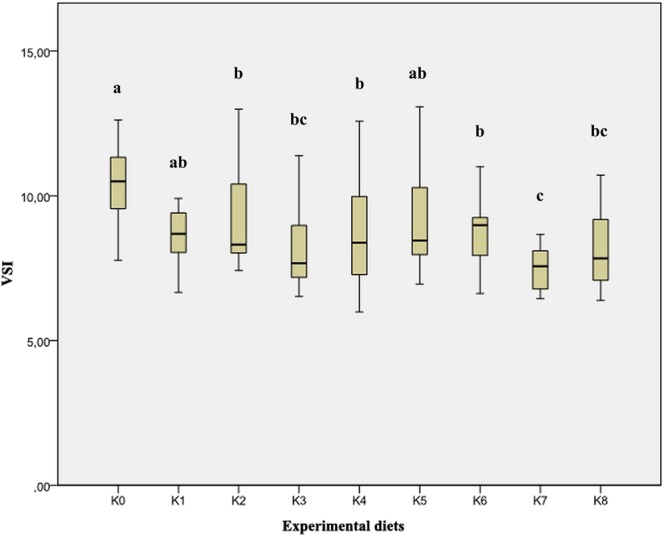
Viscerosomatic index (VSI) of fish fed control and experimental diets.

The results of the average index values of goblet cells ranged from the lowest values of 0.20 and 0.21 in groups K5 and K0, respectively. The highest value of the goblet cell density index was found in the feeding group K8 (0.86). By the examination of histological preparations of the hepatic tissue, hepatocytes irregular in shape were noticed. In general, hepatocytes showed typical chord-like arrangement surrounded by sinusoids and connective tissue. In histological preparations, the pancreatic tissue is in the form of disseminated isles in the liver tissue, surrounding blood vessels and bile ducts. Accumulation of fat in hepatocytes was not found and the emphasis was placed on glycogen stored in the liver, which was highlighted with PAS stain as a vitreous cluster in the cytoplasm and was mostly exhibited in the group K0 (Figs. 3 and 4). In the experimental group K7, infiltrating leukocytes were observed, which could indicate an initial inflammatory process (Fig. 5). Quantitative histological analysis of liver (Fig. 7) points at a significant decrease in the perimeter of the nucleus of hepatocytes in all nutritional groups when compared with the control (*p* < 0.0005). The average values of the size of the hepatocyte nucleus were lowest (between 19.95 µm and 20.04 µm) in the group K7 and highest in the group K8 (27.32 µm). In order to facilitate the interpretation of histological results, hepatosomatic (HSI) and viscerosomatic (VSI) indices were calculated and are presented in Figs. 8 and 9, respectively. The average value of HSI ranged from the highest value (3.88 ± 0.48) in the control group K0 to the lowest (2.20 ± 0.28) in the experimental group K8. The average value of VSI ranged from the highest value (10.32 ± 1.48) in the control group K0 to the lowest (7.51 ± 0.77) in the group K7 (Fig. 9).

**Figure 10:**
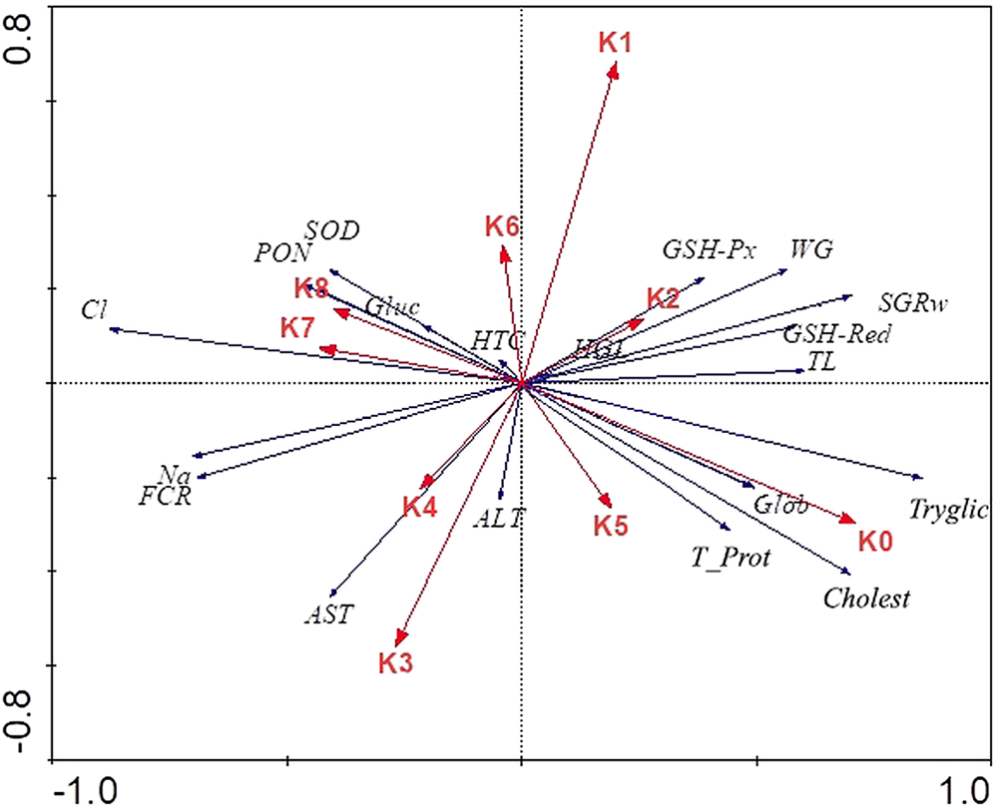
RDA analysis plot. The arrow length represents the strength of the correlation between the experimental diets and the blood and growth indicators. The longer the arrow length, the stronger the correlation. The perpendicular distance between indicators and experimental diets axes in the plot reflects their correlations. The smaller the distance, the stronger the correlation. (K0-K8 – experimental diets; TL – Total length WG – Weight gain; FCR – Feed conversion ratio; SGRw – Specific growth ratio (weight); PON – Paraoxonase 1; SOD - Superoxide dismutase; GSH-Px - Glutathione peroxidase; GSH-Red - Glutathione reductase; HTC – Hematocrit; HGL – Hemoglobin; Tryglic – Tryglicerides; Glob - Total globulins; Cholest – Cholesterol; T_Prot – Total proteins; ALT – Alanine aminotransferase; AST - Aspartate transaminase; Na – Sodium; Cl – Chloride; Gluc – Glucose).

The eigenvalues and percentage of variance explained by RDA are presented in Table 4. To visualize the relationship between experimental diets and measured indicators, RDA plot is also presented (Fig. 10).

**Table 4:** Eigenvalues and percentage of variance explained by RDA between environmental (experimental diets) and dependent variables (growth and blood parameters) (Monte Carlo test with 499 permutations; *p* < 0.05).

Axis	1	2	3	4	Total variance
Eigenvalues	0.248	0.100	0.072	0.056	1.000
Correlation between dependent and environmental variables	0.944	0.748	0.846	0.807	
**Cumulative percentage variance**					
- depended variables	24.8	34.8	42.0	47.6	
- between depended and environmental variables	44.2	62.1	75.1	85.1	
Sum of all constrained eigenvalues					1.000
Sum of all canonical eigenvalues					0.560

All four eigenvalues reported above are canonical and correspond to axes that are constrained by the environmental variables.

RDA analysis showed that different experimental diets significantly influenced growth and health parameters (Monte Carlo test with 499 permutations; *p* < 0.05). The four ordinates explained 85.1% of the total variability of results and 47.6% of the variability in the investigated parameters (Table 4). Considering the dependent variables investigated, significant differences between experimental diets K0 (F = 4.38; *p* = 0.002), K1 (F = 2.48; *p* = 0.01), K2 (F = 2.77; *p* = 0.004), K3 (F = 2.41; *p* = 0.014), K5 (F = 3.01; *p* = 0.008) and K6 (F = 1.91; *p* = 0.046) were revealed. In general, strong positive correlation was indicated for K2 with weight gain (WG) and specific growth rate (SGRw), while strong negative correlation was detected on feed conversion ratio (FCR) for the same experimental diet, which corresponds to the obtained results regarding growth parameters. Serum lipid components (cholesterol and triglycerides) were strongly correlated with the control. Higher inclusion of SBM (K3, K4) and SBM + BY (K7, K8) in experimental diets strongly affected concentrations of a biomarker for liver damage (AST) and oxidative stress-related enzymes - paraoxonase (PON) and superoxide dismutase (SOD) respectively. GSH-Px had evincive but subtle relationship with K2 while the activity of GSH-Red was significantly reduced in the groups with 50% and higher replacement of FM. (Fig. 10).

## Discussion

Diet change from FM to SBM and BY presents several metabolic and health challenges for farmed fish. When compared to the results of growth of brown bullhead promoted by vitamin C and soybean lecithin^7^, the feed conversion rate of fish in the present study was more effective. The average SGRw values were somewhat lower, compared to the^7^, which can be explained by the older fish category and the less pronounced metabolic rate of the analysed fish in the present study^26^. conducted a the long-term feeding trial (6 months) of rainbow trout (*Onchorinchus mykiss*) with the complete replacement of FM with a combination of vegetable protein and confirmed the significant difference in weight gain only after 12 weeks, thereby highlighting the importance of longer-term feeding studies. Duration of the present feeding study is considered adequate for the investigated species compared to similar research^7,56^. Growth performance retardation was detected in the fish fed 75% and 100% of FM substitution, irrespective of the inclusion of BY. Similar results were obtained by^57^ and^58^ when FM was replaced with SBM in African catfish (*Clarias gariepinus*) and cuneate drum (*Nibea miichthioides*), respectively. In contrast, the results of the research do not agree with the research performed on African catfish (*Clarias gariepinus*)^59^ and tilapia (*Oreochromis niloticus × O*. *aureus*)^60^ in which 75% of FM was successfully replaced by SBM. Juvenile common dentex (*Dentex dentex*) tolerates up to 40% replacement of FM with SBM^61^. Moreover, by replacing 10% of FM, fish obtained better growth performance compared to control. Similar effect of replacing FM with SBM was found in present study since diet K2 (50% replacement SBM) yield better growth results than control. Also, according to^29^, brewer’s yeast can replace 50% of fishmeal protein with no negative effects in sea bass (*Dicentrarchus labrax*) juveniles performance. Moreover, the inclusion of up to 30% brewer’s yeast in the diet improved sea bass feed efficiency. Due to its potential as an immunostimulant, brewer’s yeast was used in feeding trial with Indian carp (*Labeo rohita*) (1, 2 and 4%) and did not obtain significant differences in growth performance between different percentages of substitute^28^. Brewer’s yeast in present study was evaluated as a substitute for a lower percentage of inclusion (8%) and growth performance results of supplemented diets did not differ significantly with control and yeast-free diets up to 50% FM replacement. Brewer’s yeast, as a by-product of alcohol fermentation, is therefore imposed as acceptable and cost-effective SBM supplement to replace FM up to 50% in experimental diets for brown bullhead.

Various factors including differences in species, age, sex, water quality, water temperature and handling methods may contribute to variability in hematological and biochemical data that is difficult to interpret. For this reason it is difficult to compare results from different studies or set ‘normal ranges’ so determination of reference intervals for each set of culture conditions has been suggested by^24^. In this study, replacement of fish protein meal with soybean and brewer’s yeast in diet for *A*. *nebulosus* showed a hypolipidemic effect manifested by reduced concentration of serum triglycerides and cholesterol. Similarly, SBM caused decrease in serum lipids in rice field eel *Monopterus albus*^62^ Significant increase in AST activity provides the evidence of liver tissue damage and increased amino acids transamination process, which was also reported by^9^ during the study in which soybean proteins were included into the diet of Nile tilapia, *Oreochromis niloticus*. A decline in serum globulin may be due to reduced protein synthesis or a protein breakdown caused by cirrhosis, intestinal mucosal disruption, which resulted from high levels of SBM in the diet^63^. Increased concentrations of sodium and chloride may cause osmoregularity issues in fish, and are usually a symptom of malnutrition and liver injury. Recent study had indicated that oxidative damage was often accompanied by the reduction of antioxidant capacity due to antinutrient from soy protein in fish feed such as β-conglycinin^64^. Our study demonstrated that the activity of GSH-Red was reduced in the groups fed with diets with higher replacement of FM with SBM and BY. Thus, in this study, the decrease in GSH-Red activity may contribute to the prevalence of inflammatory stages in the liver and intestines. Moreover, our results showed that SBM alone or in combination with brewer’s yeast increased protective activities of SOD in blood as a form of a first attempt to overcome scavenging process of oxidation induced by SBM. As an antioxidant, SOD mainly exists in cell metabolism, playing a protective role in cells by the disproportionation of toxic free radicals to inactive hydrogen peroxide and oxygen molecules^65^. In a study of prolonged starvation in common dentex (*Dentex dentex*)^27^ the activity of the antioxidative enzymes superoxide dismutase (SOD) was significantly increased, moreover the prooxidative activity for the main flavonide present in soy meal was demonstrated *in vitro* study^66^, therefore increased SOD activity in our study, in groups with higher percentages of SBM as replacement for FM, could be as a results from oxidative stress conditions caused with undernutrition and antinutritional agents. When using high dietary levels of plant derived materials, particularly those derived from soybean, it is important to consider the impacts on gut histology as the gastrointestinal tract can be an important infection route for some pathogens in fish^67^. Thus, the aim of the present study was to investigate the possible effects of SBM and BY feed on gut epithelial and hepatic cell histology. Furthermore, these effects were evaluated through qualitative and quantitative histological approach. Unlike common carp and salmon species where the intestinal folds are similar in length and regularly lined within the intestinal lumen with uniformed hepatocytes, the results of histological analysis of omnivore brown bullhead showed species diversity of the intestinal folds, which were more branched and completely filled the intestinal lumen, with hepatocytes irregular in shape. Furthermore, finding bile ducts and blood vessels surrounded by pancreatic tissue also falls into the normal formation of hepatopancreas^68^.

Evaluation of histological structure of digestive organs in fish fed plant and yeast ingredients provides valuable information about digestive capacity and potential health benefits of new diets^69^, and it was suggested as a method of choice in diet trials^39^. Histopathological estimates may vary depending on the species and feed used in the experiments, and there have been numerous investigations regarding evaluation of the feasibility of FM replacements in aquaculture species like European catfish^56^, seabass^21,38^, silvery-back porgy^48^, Atlantic salmon^70^, rainbow trout^71^, common carp^72^ and orange-spotted grouper^73^. Although, the evidence from available literature concerning FM replaced with SBM in aquaculture exists, morphologic studies addressing the effect of nutritional substitutions on the health status of fish’s liver and intestinal barrier are still lacking^74^, especially in Ictalurid species where only research with yeast was applied^31,32^. Because of its anti-nutritional properties, a high proportion of FM replaced with SBM in some fish species may cause pathological changes of intestinal mucosa described as “non-infectious subacute enteritis of distal intestine”^75^. This pathological occurrence is associated with shortening of intestinal cells (villa and microvilli), infiltration and thickening of the connective tissue (*lamina propria*) inflammatory cells and alterations in the structure of enterocytes^21,38,76,77^. These morphological changes are responsible for the retardation in the growth of certain fish species when feeding them diet containing SBM^15,19,20^. High levels of dietary soybean products may affect intestinal integrity. Damage is often characterised by changes in number of mucus-producing goblet cells^30^, intracellular absorptive vacuoles^38^, cellularity of *lamina propria*, amount of connective tissue, and degrees of mucosal folding and infiltration of the epithelium or *lamina propria* by inflammatory cells^21^. Some researchers emphasised that enteritis is dependent on the percentage of FM replaced with plant proteins (mostly SBM) in fish feed^14,78^, which corresponds to our results and replacement ratios.

The goblet cells, present along the entire intestine, are responsible for the synthesis and secretion of the protective mucus layer that covers the epithelial surface. This mucus layer acts as a medium for protection, lubrication and transport between the luminal contents and the epithelial lining, and it is an integral structural component of the intestine^79^. In our study, modest changes have been found in the mucosa of fish fed diets of higher replacement ratios (increased mucosal epithelium and accumulation of mucus) and there were indications of inflammation in fish fed diets where FM was completely replaced with SBM, regardless of the inclusion of BY (increased number and size of goblet cells, thickening of *lamnia propria*) (Figs. 1 and 2). An increased production of goblet cells during carp fasting was indicated by^80^, while^16^, by addition of β-(1,3)-glucan from yeast cells, did not find any improvement of morphological changes of intestinal mucosa caused by the major inclusions of SBM in the diet of Atlantic salmon. The leucocytes in *lamina propria* interact with epithelial cells and protect the re-absorptive epithelia by killing infected cells and attracting other immune cells, such as eosinophilic granular cells, to combat infection. Previous works have suggested that SBM^20^ and yeast^32^ added to fish feed could increase goblet cell population. In our study, these results have been confirmed and it was concluded that a higher number and production of mucous from these cells are an adaptive mechanism of guts toward protection of the re-absorptive epithelium from antinutritive factors in soy beans. Also, in the present study, an increase of the population of leucocytes was observed in the areas of gut *lamina epithelialis* and *lamina propria* in fish fed a higher share of SBM diets, which combat tissue inflammation already present. On the other hand, general histopathological analysis showed the undisturbed, tightly bounded enterocyte architecture in the intestines of the groups K0, K1, K5 and K6, which is of great importance since expanded intercellular spaces between enterocytes facilitate the entrance of the potential pathogens in the bloodstream^81^. In the experiment with Egyptian sole (*Solea aegyptiaca)*, 30% of FM was replaced with SBM without pathological changes in the intestines observed^82^. A short inflammatory reaction to the inclusion of SBM in carp was demonstrated by^19^. After a period of one-month adaptation to feed, intestines restore their normal histological structure, which was not the case in the presented brown bullhead research. Signs of enteritis when FM was substituted with SBM in the diets of sunshine bass was noted by^83^, but no significant differences in gut integrity were observed. The appearance of teleost hepatocytes can vary greatly due to sex, maturity, diet, season, contaminant exposure and other factors^84^. Histological changes of the fish’s liver are easily recognizable if inadequate fish feed is used^85^. The most common observable changes include: vacuolization of hepatocytes, fatty degeneration, changes in metabolic activities, changes in liver parenchyma and necrosis^68^. Along with reduced somatic indices of the liver^86^, observed a slight hypertrophy of hepatocytes in turbot fed a higher percentage of rapeseed protein isolate. In contrast, in the control diet and lower percentages of replacement, they noticed a pronounced hypertrophy and reduced sinusoidal space. The present study demonstrated that diets with higher share of SBM and a combination of SBM and yeast increased the tissue reaction of liver in the light of hepatocyte nuclei apoptosis (Figs. 3 and 4). Although fish exhibited hepatocyte nucleus morphology disruption and indication of kariopicnosis, no mortalities occurred at the end of the experiment. Liver apoptosis is a proven indicator of malnutrition, both in mammals and fish^87^. A picnotic hepatocytes and fatty degeneration was found by^72^ when feeding common carp diets in which FM was partly or fully replaced with fat-extracted SBM. Within our study, along with an initial decrease in the size of hepatocyte nucleus, which is a characteristic of liver apoptosis (Fig. 7), hepatosomatic indices (HSI) were significantly reduced in all feeding groups (Fig. 8) when compared with the control. This corresponds to liver hypotrophy of brown bullhead fed SBM and BY. HSI and VSI indicate the status of energy reserves in fish while its lower values are often caused by poor environmental conditions, diseases and inadequate nutrition^88^. VSI and HIS results were slightly lower in present study when comparing to^7^ possibly due to the difference in the fish size. Reduced HSI and VSI can be partly explained by lower protein and lipid content in the experimental diets and higher percentages of FM replacement. Although final weight, SGR and FCR were unaffected in sharpsnout seabream (*Diplodus puntazzo*) when fed partial fish meal substitution by soybean meal (40% replacement), values of HSI and VSI were found significantly lower^89^. As^36^ in their study proposed histopathological approach, using fish intestine as a sensitive bioindicator organ of pollution impact in the freshwater ecosystem, in present study morphological changes in liver and intestines are propounded as a biomarker tool for integrating cumulative effects of different FM replacements. Furthermore, moderate leucocyte infiltration present in the feeding group K7, which coincides with the lowest HSI and hepatocyte nuclei size, certainly indicates serious liver damage due to antinutritive substances present in a higher share of soybean in the diet of brown bullhead (Fig. 5). Impaired liver indices (HSI) were further linked with histochemical analysis of glycogen reserves, which were diminished in animals fed a higher percentage of FM replacement and can implicate fast onset and evolution of liver metabolic stress because glycogen deposits in the liver were utilized to maintain metabolism. A lower vacuolization as an indicator of malnutrition and hunger in trout fed concentrated tomato protein was described by^90^, since glycogen and lipids in the liver used for maintenance of metabolism were depleted. Taken together, both ways of liver analyses indicated metabolic disorder and liver injury in brown bullhead fed a higher share of soybean protein. Viscerosomatic indices (VSI) were lower in all diet groups when compared to the control diet (Fig. 9), which could be due to the lower lipid content in all experimental feeds and subsequently lower lipid accumulation in the viscera, as it was suggested by^91^ after replacing cod liver oil with palm oil in the feed of juvenile Nile tilapia (*Oreochromis niloticus*). Although isoenergetic, the relative difference in the lipid content of the diets can slightly affect the evaluation of FM replacement so it must be taken into consideration as a possible research limitation. Further work on this particular topic (fatty acid profile, amino acid evaluation) should yield interesting information concerning fish growth performances and meat quality. The present study, together with future investigations into this topic, will give a wider vision to the usefulness of FM replacement in the farming of brown bullhead. To optimise expenses in the aquaculture of brown bullhead, future investigations should be focused on a mixture of SBM with various plant protein sources in combination with BY.

## Conclusions

The growth performance of fish fed diets up to 50% of FM replacement did not deviate significantly from the control. Moreover, 50% replacement of FM with SMB indicated the highest growth performance values in fish. A marked hypolipidemic activity of SMB and brewer’s yeast as dietary components was observed. Increased AST activity suggested liver tissue damage in higher FM replacement with SBM. Additionally, high dietary SMB exerted oxidative stress in fish and activation of antioxidative defence mechanisms. Morphological analysis of the digestive system of fish shows significantly lower results of the fish fed diets 75% and 100% of FM replacement compared to the control. In addition, the results reinforce the high potential of histopathological assessment in both qualitative and quantitative manner to estimate fish health during FM replacement trials. Introduction of higher amounts of SBM used in the research induced enteritis and apoptotic reaction in the liver cells of fish. Brewer’s yeast was indicated as acceptable and cost-effective SBM supplement to replace FM up to 50% in experimental diets for brown bullhead. To reduce the risk of SBM-induced enteritis and liver lesions in the aquaculture of brown bullhead, the usage of partial replacement of FM with SBM of up to 50% has been recommended.
