# Author Correction: Growth, health aspects and histopathology of brown bullhead (*Ameiurus nebulosus* L.): replacing fishmeal with soybean meal and brewer’s yeast

**DOI:** 10.1038/s41598-020-67558-6

**Published:** 2020-07-01

**Authors:** Daniel Matulić, Josip Barišić, Ivica Aničić, Tea Tomljanović, Roman Safner, Tomislav Treer, Jian Gao, Ines Glojnarić, Rozelindra Čož-Rakovac

**Affiliations:** 1 Department of Fisheries, Apiculture, Wildlife Management and Special Zoology, Faculty of Agriculture, University of Zagreb, Zagreb, Croatia; 2 Laboratory for Biotechnology in Aquaculture, Ruđer Bošković Institute, Zagreb, Croatia; 3 College of Fisheries, Huazhong Agricultural University, Wuhan, China; 4 Fidelta Ltd., Zagreb, Croatia

Correction to: *Scientific Reports* 10.1038/s41598-020-57722-3, published online 24 January 2020

This Article contains errors.

In the Introduction, the sentence,

“Economic benefits from aquaculture occur primarily in Chile, China, Bulgaria and Belarus^43,44,45,46^ although the magnitude of these benefits remains uncertain”

should be removed.

The RDA analysis reported in the paper is incorrect. An updated Fig. 10 and Table 4 appear as Fig. 1 and Table 1 respectively.

**Figure 1:**
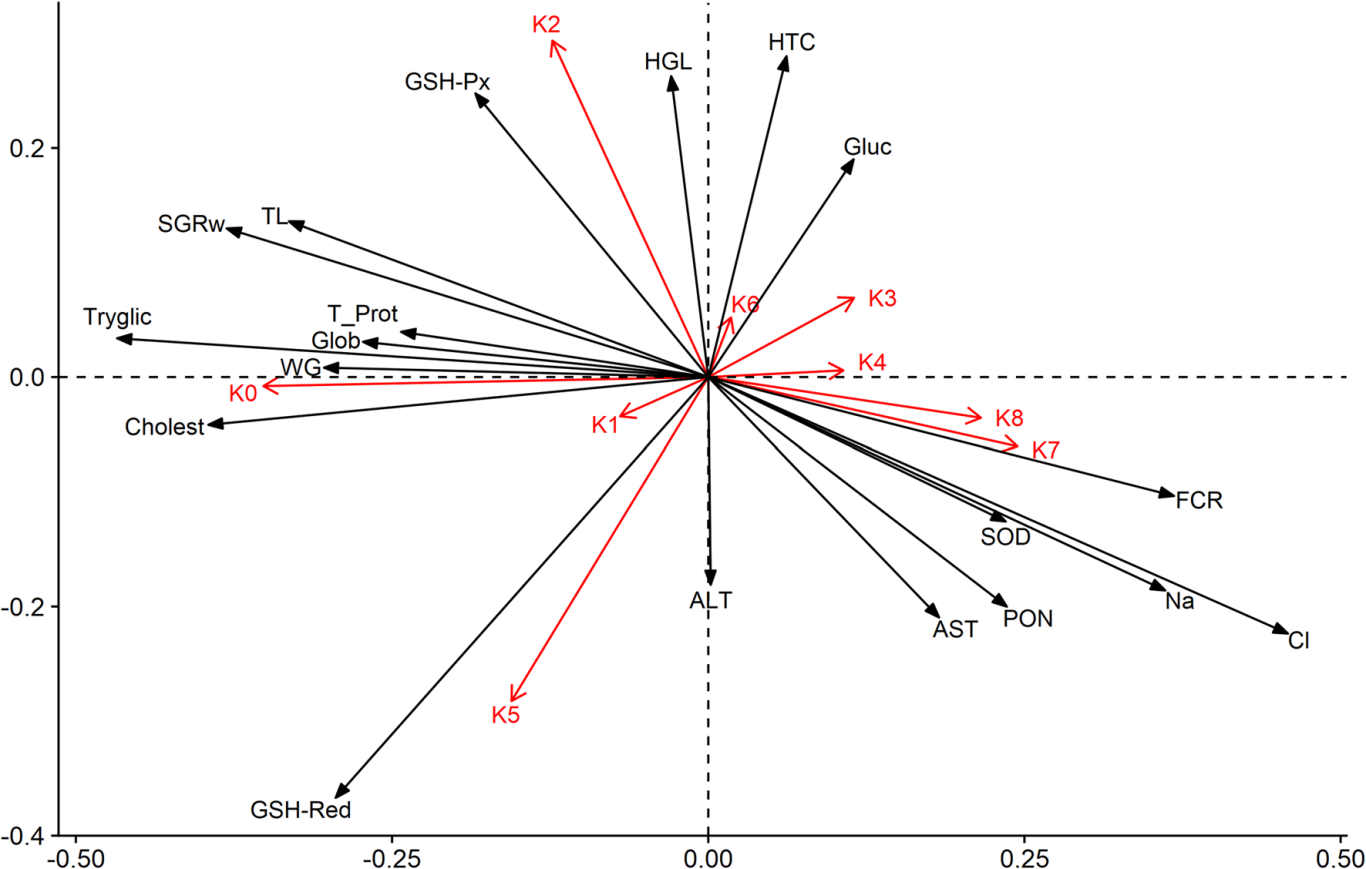
RDA analysis plot. The arrow length represents the strength of the correlation between the experimental diets and the blood and growth indicators. The longer the arrow length, the stronger the correlation. The perpendicular distance between indicators and experimental diets axes in the plot reflects their correlations. The smaller the distance, the stronger the correlation (K0–K8—experimental diets; TL—total length; WG—weight gain; FCR—feed conversion ratio; SGRw—specific growth ratio (weight); PON—Paraoxonase 1; SOD—superoxide dismutase; GSH-Px—glutathione peroxidase; GSH-Red—glutathione reductase; HTC—hematocrit; HGL—hemoglobin; Tryglic—tryglicerides; Glob—total globulins; Cholest—cholesterol; T_Prot—total proteins; ALT—alanine aminotransferase; AST—aspartate transaminase; Na—sodium; Cl—chloride; Gluc—glucose).

**Table 1:** Eigenvalues and percentage of variance explained by RDA between environmental (experimental diets) and dependent variables (growth and blood parameters) (Monte Carlo test with 499 permutations; *p* < 0.05)

Axis	1	2	3	4	Total variance
Eigenvalues	0.265	0.096	0.074	0.068	1.000
Correlation between dependent and environmental variables	0.938	0.826	0.64	0.741	
Cumulative percentage variance					
Dependent variables	26.5	36.1	43.5	50.3	
Between dependent and environmental variables	46.7	63.5	76.5	88.4	
Sum of all constrained eigenvalues					1.000
Sum of all canonical eigenvalues					0.569

All four eigenvalues reported above are canonical and correspond to axes that are constrained by the environmental variables.

In the Material and Methods, the sentence,

“Canoco 4.5.5 for Windows was used for the analysis^55^ according to^54^”.

should read:

“R statistical software (v.3.6.2, R Foundation for Statistical Computing, Austria) was used for the analysis, with implemented Community Ecology Package (VEGAN)^1^ for Canonical Analysis as suggested by^2^”.

In the Results, under the subheading ‘Tissue morphology’,

“RDA analysis showed that different experimental diets significantly influenced growth and health parameters (Monte Carlo test with 499 permutations; *p* < 0.05). The four ordinates explained 85.1% of the total variability of results and 47.6% of the variability in the investigated parameters (Table 4). Considering the dependent variables investigated, significant differences between experimental diets K0 (F = 4.38; *p* = 0.002), K1 (F = 2.48; *p* = 0.01), K2 (F = 2.77; *p* = 0.004), K3 (F = 2.41; *p* = 0.014), K5 (F = 3.01; *p* = 0.008) and K6 (F = 1.91; *p* = 0.046) were revealed. In general, strong positive correlation was indicated for K2 with weight gain (WG) and specific growth rate (SGRw), while strong negative correlation was detected on feed conversion ratio (FCR) for the same experimental diet, which corresponds to the obtained results regarding growth parameters. Serum lipid components (cholesterol and triglycerides) were strongly correlated with the control. Higher inclusion of SBM (K3, K4) and SBM + BY (K7, K8) in experimental diets strongly affected concentrations of a biomarker for liver damage (AST) and oxidative stress-related enzymes—paraoxonase (PON) and superoxide dismutase (SOD), respectively. GSH-Px had evincive but subtle relationship with K2 while the activity of GSH-Red was significantly reduced in the groups with 50% and higher replacement of FM (Fig. 10)”.

should read:

“RDA analysis showed that different experimental diets significantly influenced growth and health parameters (Monte Carlo test with 499 permutations; *p* < 0.05). The four ordinates explained 88.4% of the total variability of results and 50.3 % of the variability in the investigated parameters (Table 4). Considering the dependent variables investigated, significant differences between experimental diets K0 (F = 4.11; *p* = 0.004), K1 (F = 2.65; *p* = 0.01), K2 (F = 3.12; *p* = 0.002), K3 (F = 2.18; *p* = 0.032), and K5 (F = 3.23; *p* = 0.002) were revealed. In general, positive correlation was indicated for K0, K2 and K5 with weight gain (WG) and specific growth rate (SGRw), while strong negative correlation was detected on feed conversion ratio (FCR) for the same experimental diet, which corresponds to the obtained results regarding growth parameters. Serum lipid components (cholesterol and triglycerides) were strongly correlated with the control. Diets K1, K3, K4 along with K6 weren’t indicated as multivariate influential while higher inclusion of SBM + BY (K7, K8) in experimental diets affected concentrations of a biomarker for liver damage (AST) and oxidative stress-related enzymes—paraoxonase (PON) and superoxide dismutase (SOD), respectively. GSH-Px had relationship with K2 while the activity of GSH-Red was significantly reduced in the groups with 50% and higher replacement of FM with SBM (Fig. 10)”.
